# Evaluation of treatment plans using various treatment techniques for the radiotherapy of cutaneous Kaposi's sarcoma developed on the skin of feet

**DOI:** 10.1120/jacmp.v15i6.4970

**Published:** 2014-11-08

**Authors:** Jong Min Park, Il Han Kim, Sung‐Joon Ye, Kyubo Kim

**Affiliations:** ^1^ Department of Radiation Oncology Seoul National University Hospital Seoul Korea; ^2^ Institute of Radiation Medicine Seoul National University Medical Research Center Seoul Korea; ^3^ Biomedical Research Institute Seoul National University College of Medicine Seoul Korea; ^4^ Center for Convergence Research on Robotics Advance Institutes of Convergence Technology Suwon Korea; ^5^ Department of Radiation Oncology Seoul National University College of Medicine Seoul Korea; ^6^ Program in Biomedical Radiation Sciences, Department of Transdisciplinary Studies Seoul National University Graduate School of Convergence Science and Technology Suwon Korea

**Keywords:** cutaneous Kaposi's sarcoma, volumetric‐modulated arc therapy, high‐dose‐rate brachytherapy, Freiburg flap applicator

## Abstract

The purpose of this study was to investigate the plan qualities of various treatment modalities for the radiotherapy of cutaneous Kaposi's sarcoma developed on the skin of the foot. A total of six virtual targets were generated on the skin of the foot in CT images. Five types of treatment plans were generated using photon beams (PB), electron beams (EB), high‐dose‐rate (HDR) brachytherapy with a Freiburg flap applicator, intensity‐modulated radiation therapy (IMRT), and volumetric‐modulated arc therapy (VMAT) techniques. Plans for each of the six targets (single‐target plans) and also for the combined target consisting of the six single targets combined (multitarget plans) were generated. Dose‐volumetric analysis was performed for the targets and normal tissues. The averaged conformity index (CI) and homogeneity index (HI) values for each single target using PB, EB, HDR, IMRT, and VMAT techniques were 1.97, 2.39, 1.60, 4.60, and 0.80 and 1.05, 1.11, 1.52, 1.04, and 1.04, respectively. For the multitarget, the CI values were 3.99, 5.08, 1.38, 1.95, and 0.84, and the values of HI were 1.10, 1.36, 1.43, 1.06, and 1.04, respectively. The averaged mean doses to normal tissue were 2.5, 2.7, 3.6, 1.7, and 2.9 Gy for single‐target plans, and 21.3, 14.6, 14.2, 14.3, and 13.0 Gy for the multitarget plans, respectively. The VMAT demonstrated dosimetric advantages and better treatment efficiency over other techniques for the radiotherapy of multifocal skin disease of the feet.

PACS number: 87.55.dk

## I. INTRODUCTION

Kaposi's sarcoma (KS), originally described by Moritz Kaposi in 1872, is a rare form of vascular malignant skin disease of the extremities which may spread to the viscera.^(1)^ The epidemic form of KS is frequently demonstrated in patients infected by human immunodeficiency virus.^(2)^ Even though the percentage of acquired immune deficiency syndrome (AIDS) patients with KS is decreasing, the overall incidence of epidemic KS is increasing along with the rising prevalence of AIDS.^(3)^ It has been well established that KS is responsive to radiation therapy, and in response various radiation treatment schemes have been studied.^1^, ^2^, ^3^, ^4^, ^5^, ^6^, ^7^, ^8^, ^9^, ^10^, ^11^, ^12^, ^13^ The response rate of KS in epidemic form to radiation therapy has been demonstrated to be as high as 90%, and it has been proven that radiation therapy shows better symptomatic control than chemotherapy.^(14)^

For KS developed at extremities, a modality for radiation therapy was suggested that utilizes two opposing megavoltage photon beams while immersing the extremities in a water reservoir.^(1)^ This study demonstrated that total disappearance of the skin lesions was achieved in 89% of the patients; however, the limb edema regressed completely in only 56% of the patients^(1)^ due to reduced lymphatic drainage.^(15)^ In order to relieve edema, electron beam therapy could be implemented as an alternative treatment for extremity KS. For cutaneous lesions, electron beam therapy is considered to be effective in the sparing of normal tissue underlying skin due to the limited penetrating power of electrons.^(16)^ A possible disadvantage of electron beam therapy is the necessity of a large number of treatment fields because KS is a multifocal disease.^(11)^ This could result not only in the complicated positioning of patients, leading to very long treatment times, but also a risk of under‐ or overirradiation of the skin due to convexity of the target, or when field matching is needed.^(15)^ A further treatment modality for skin cancers is high‐dose‐rate (HDR) brachytherapy with surface applicators such as Leipzig, Valencia, and Freiburg flap applicators (FF applicator).^17^, ^18^, ^19^, ^20^, ^21^ However, Leipzig and Valencia applicators can only be used for the treatment of skin cancers covering areas smaller than 3 cm in diameter^19^, ^20^, ^21^ and are, thus, unsuitable for treatment of extremity KS. Due to its flexibility and ability to cover large areas, the FF applicator could be a candidate for the treatment of extremity KS.^17^, ^18^ Limitations of HDR brachytherapy with an FF applicator include the need for thick boluses to compensate for the lack of backscatter material on top of the applicator, and the air between the spheres.^(17)^ Furthermore, HDR is generally more labor‐intensive than external beam therapy, which could be problematic in busy institutes with limited resources. Nicolini et al.^(15)^ proposed volumetric‐modulated arc therapy (VMAT) with a natural wax bolus on the extremities as a treatment modality for extremity KS. They compared VMAT plans for lower extremities with electron beam plans and concluded that VMAT was superior to electron beam therapy.

Although cutaneous KS developed at extremities has been studied, no study thus far has been performed for the treatment of cutaneous KS developed specifically on the skin of the foot. Due to the unique geometry of the foot, it is relatively difficult to apply electron beam (EB) therapy or a tangential photon beam (PB) to the cutaneous disease at this site. In addition, no study has been performed yet to compare external beam therapy vs. HDR with FF applicator for cutaneous KS of the foot or at extremities.

In this study, various treatment techniques have been compared for the radiotherapy of cutaneous KS developed on the skin of foot. Treatment plans using photon beam (3D conformal radiation therapy, 3D CRT), electron beam, HDR with FF applicator, intensity‐modulated radiation therapy (IMRT), and VMAT techniques were compared in terms of dose‐volumetric quality of the treatment plan.

## II. MATERIALS AND METHODS

### A. Simulation and contouring

**Figure 1:**
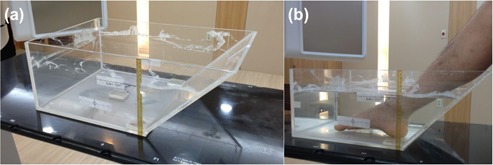
The custom‐made water reservoir for the skin treatment of the foot (a) and the patient setup using this water reservoir filled with water (b). The reservoir is made of acrylic and the level of water surface is marked. There is a slope on one side of the reservoir for the comfortable setup of patients when they put their foot inside the reservoir. The water reservoir could eliminate the buildup effect at cutaneous regions, thereby enabling the prescribed dose to be delivered to the target located at a superficial region.

**Figure 2:**
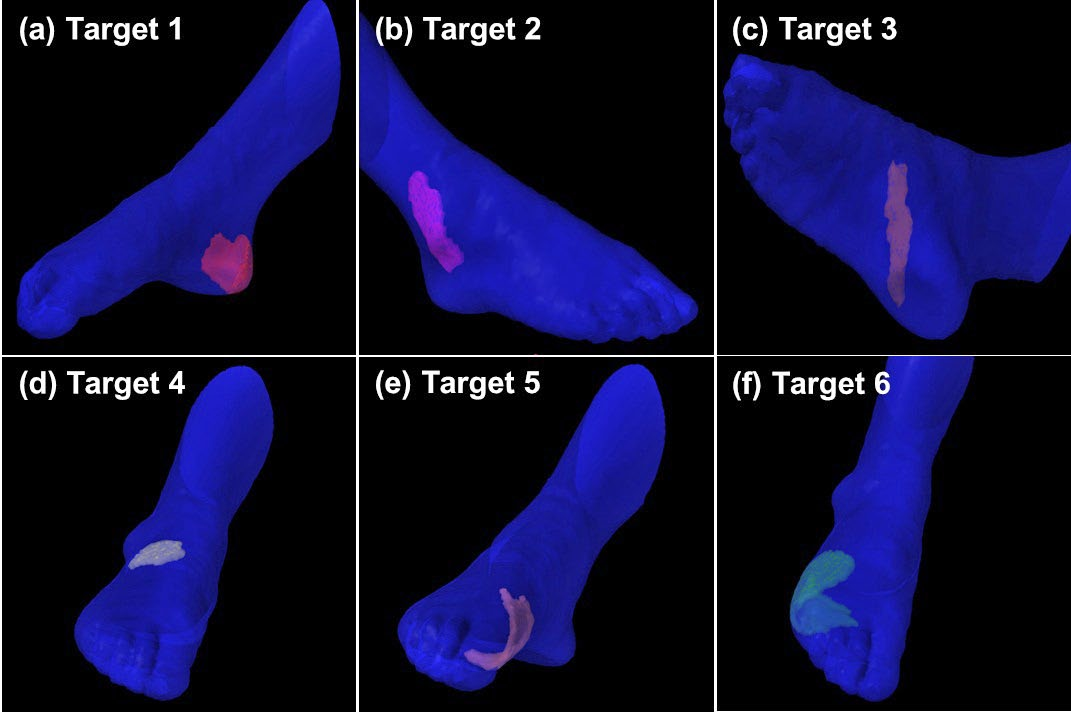
The virtual clinical target volumes (CTVs) located at cutaneous regions are illustrated. A total of six targets on the skin of the foot are postulated for the treatment planning of cutaneous Kaposi's sarcoma. Targets 1 to 6 is defined at heel pad (a), inner ankle (b), inner arch (c), dorsal surface (d), inner big toe mound (e), and outer little toe mound (f) of foot. The thickness of target is 5 mm and the area of target 1 to 6 is 30.8, 20.6, 13.0, 9.4, 19.8, and $51.8\;{\text{cm}}^{2}$, respectively. Each single target is summed and postulated for the treatment planning of multifocal Kaposi's sarcoma. The total area of multifocal target is $145.4\;{\text{cm}}^{2}$.

After an institutional review board (IRB) approval, computed tomography (CT) images of the corresponding author's foot were acquired using Brilliance Big Bore CT (Philips, Amsterdam, Netherlands). The right foot was immersed in a water reservoir specially manufactured for radiotherapy of the skin of foot to eliminate the buildup effect of the megavoltage photon beam. The CT images covering the whole volume of the foot were taken with a slice thickness of 3 mm. The water reservoir and the setup of the patient are shown in Fig. 1. A total of six virtual clinical target volumes (CTVs) were delineated on the acquired CT images. The virtual CTVs were defined at the heel pad (target 1), inner ankle (target 2), inner arch (target 3), dorsal surface (target 4), inner big toe mound (target 5), and outer little toe mound (target 6) of the foot (Fig. 2). The thickness of each target was 5 mm and the areas of targets 1 to 6 were 30.8, 20.6, 13.0, 9.4, 19.8, and $51.8\;{\text{cm}}^{2}$, respectively. Planning target volumes (PTVs) were generated with a margin of 5 mm around the CTVs. The whole volume of the foot minus the volume of each of the six targets was defined as the foot organ at risk (OAR). The foot OAR was contoured with a margin of 5 cm from the PTVs. Normal skin was defined over the whole volume of the foot by excluding the target volume from the skin of which thickness was 1 mm. Similarly, bone was contoured over the whole volume of the foot. Both normal skin and bone were also contoured with a margin of 5 cm from the PTVs.

### B. Planning for a single target using different radiation therapy modalities

**Figure 3:**
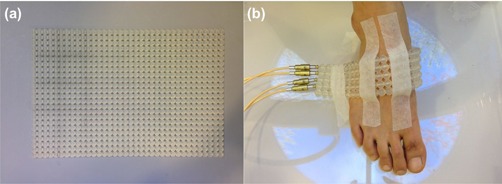
The Freiburg flap applicator (a) and its application on the skin of foot (b) are shown. It is used for the radiation therapy of skin combined with high‐dose‐rate (HDR) brachytherapy. The Freiburg flap applicator consists of a flexible mesh style surface mold formed by 0.5 cm radius silicon spheres. The spheres are tunneled through the center with flexible catheters for the positioning of Ir‐192 sources. The design of the Freiburg flap applicator keeps the distance from source to skin constant.

In order to simulate the treatment of a single‐focal skin disease, treatment plans for each target were generated. Treatment plans using external beams were generated with Eclipse system (Varian Medical Systems, Palo Alto, CA). For HDR brachytherapy planning, Oncentra system (Nucletron, BV, Veenendaal, The Netherlands) was used. For the planning of PB, IMRT, and VMAT, CT images of the foot immersed in the water reservoir were used to eliminate the buildup effect at the skin region. However, for the planning of EB and HDR, it was not possible to set up using the water reservoir with the electron applicator or FF applicator. Therefore, the water reservoir in CT images was eliminated by assigning the CT numbers of the water reservoir to −1000 in TPS. After that, for EB planning, to eliminate the buildup effect, a 1 cm thick bolus was attached to the foot. The FF applicator and the treatment setup using it are shown in Fig. 3. TrueBeam STx (Varian Medical Systems) equipped with a high definition multileaf collimator (HD MLC) was used for plans using external beams, while microSelectron (Nucletron, BV, Veenendaal, The Netherlands), with an Ir‐192 source with activity of 3.3 Ci, was used for HDR planning. The IMRT and VMAT plans were optimized with the dose‐volume optimizer algorithm (DVO, version A10) and progressive resolution optimizer algorithm (PRO3, version A10), respectively. Dose calculations for PB, IMRT, and VMAT plans were performed using the anisotropic analytic algorithm (AAA, version A10) at a grid size of 2.5 mm. The electron Monte Carlo algorithm (eMC, version A10) was used for dose calculations for EB plans at a grid size of 2.5 mm. The accuracy and the accuracy limit for eMC were set to 2 and 3, respectively. The three‐dimensional Gaussian was used for eMC calculation as a smoothing method with the smoothing level set to medium. American Association of Physicists in Medicine (AAPM) Task Group 42 (TG‐42) protocol for Oncentra (version 4.1.2.10) was used for the dose calculation of HDR plans, and no inhomogeneity correction was applied. The prescription dose was 30 Gy with a daily dose of 3 Gy. Since this was a comparative study, the same prescription was applied to all plans with different treatment techniques. For PB, IMRT, and VMAT plans, the PTVs were assigned as targets considering of setup errors, while the CTVs were assigned as targets for EB and HDR plans. Since the patient setup for EB is performed before delivery of each field by matching the KS on the skin to the field light through visual inspection, the CTVs were assigned as targets. In the case of HDR, the FF applicator was attached through visual inspection of KS on the skin by a radiation oncologist before each treatment; therefore, the CTVs were assigned as targets for HDR. However, in the cases of PB, IMRT, and VMAT, several fields are delivered sequentially after patient setup; thus, PTVs were assigned as targets for those techniques. For PB, IMRT, and VMAT plans, 6 MV photon beams were used, while for EB plans, electron energies were determined considering the target depth to achieve enough coverage for each single target. We set the plan goals for targets such that 95% of the prescribed dose covered at least 95% of the target volume. Two tangential opposing photon beams were used for both PB plans and IMRT plans. Two full arcs were used for VMAT plans. For EB plans, a single electron beam field was used. For HDR with the FF applicator, the number of channels was varied according to the area of each single target.

### C. Planning for multitarget using different modalities

A total of six targets from single‐target planning were summed and considered as one target for multitarget planning, simulating multifocal skin disease such as cutaneous KS. Treatment plans using EB and HDR technique were generated by the summation of every single‐target plan. However, for PB, IMRT, and VMAT plans, new plans covering the whole target volume were generated since the dose distributions of each single plan affected one another, causing undesired high‐dose irradiation. The four‐box technique with an MLC block, which was fit to the target shape, was used for the PB plan. A total of seven equiangular fields were used for the IMRT plan and two full arcs were used for the VMAT plan. The EB plan for the multitarget required a complicated setup with various isocenters and gantry angles, while the PB, IMRT, and VMAT plans had a relatively simple setup with a single isocenter.

### D. Dose‐volumetric analysis

The homogeneity index (HI) and the conformity index (CI) were calculated for the targets.^(22)^ The HI was calculated as follows:

(1) $$\text{Homogeneity}\;\text{Index}=D_{5\%}/D_{95\%}$$

where $D_{n\%}$ is the minimum dose delivered to n% volume of the target. The CI was calculated as follows:

(2) $$\text{Conformity}\;\text{Index}=V_{95\%}/(\text{Volume of target})$$

where $V_{n\%}$ is the volume which receives n% of prescribed dose.

Values of CI and HI close to unity indicate superior conformity and homogeneity, respectively. The maximum, minimum, and mean doses to target were calculated for all plans of different modalities. The target volumes receiving more than $105\%\;(V_{105\%})$ and $120\%\;(V_{120\%})$ of the prescription dose were also calculated.

For dose‐volumetric analysis of normal tissue, the mean doses to the foot OAR were calculated. The gradient measure (GM)^(23)^ is defined as follows:

(3) $$\text{Gradient}\;\text{Measure}=r_{50\%}-r_{100\%}$$

where $r_{n\%}$ is the equivalent sphere radius of the n% of the prescription isosodose.

The equivalent sphere is a sphere with the same volume as one inside a given isodose surface. A smaller value of GM indicates higher dose gradients around the target. From the DVHs of the foot OAR, $D_{50\%}$, $V_{20\text{Gy}}$, and $V_{10\text{Gy}}$ were calculated. The mean dose and the maximum dose to the skin and bone were also calculated. The integral doses (multiplication of the whole volume of the foot with the mean dose of the foot) were also calculated.

## III. RESULTS

### A. Dose‐volumetric results for single target

**Figure 4:**
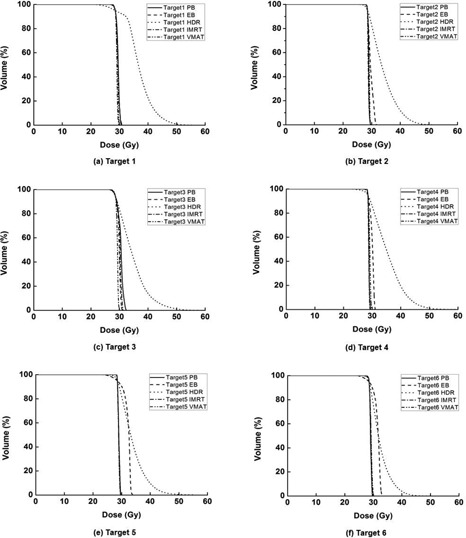
Dose‐volume histograms (DVHs) of target 1 (a), target 2 (b), target 3 (c), target 4 (d), target 5 (e), and target 6 (f) are shown. For each target, DVHs from plans using tangential photon beam (PB), electron beam (EB), high‐dose‐rate (HDR) brachytherapy with surface applicator, intensity‐modulated radiation therapy (IMRT), and volumetric‐modulated arc therapy (VMAT) are shown. The VMAT shows better target coverage and homogeneity than the other techniques.

**Figure 5:**
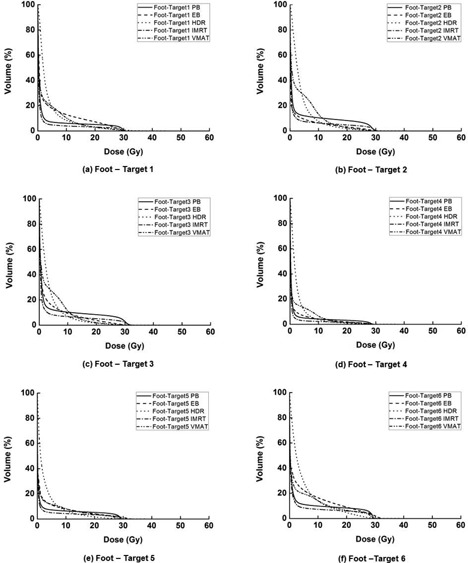
The dose‐volume histograms (DVHs) of the foot subtracted by the target (foot organ at risk, foot OAR) from the treatment plan for target 1 (a), target 2 (b), target 3 (c), target 4 (d), target 5 (e), and target 6 (f) are shown, respectively. For each target, DVHs from plans using tangential photon beam (PB), electron beam (EB), high‐dose‐rate (HDR) brachytherapy with surface applicator, intensity‐modulated radiation therapy (IMRT), and volumetric‐modulated arc therapy (VMAT) are shown. The doses delivered to the foot OAR in high‐dose regions are higher than the others when using the PB technique, and lower in the low‐dose regions than the other techniques. In the case of the HDR plans, the doses in the low‐dose region tend to be higher than the other techniques, while in high‐dose region, those are lower than the others.

**Figure 6:**
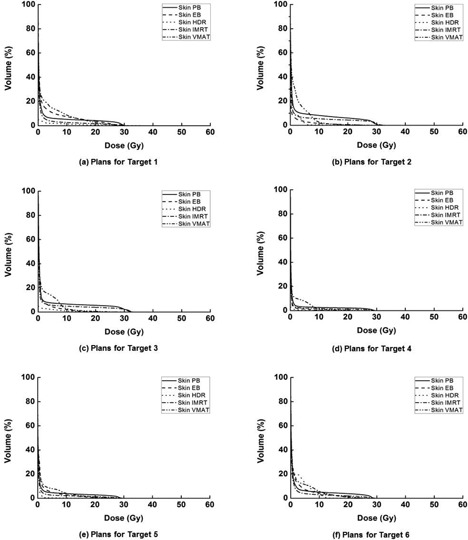
The dose‐volume histograms (DVHs) of the skin from the treatment plan for target 1 (a), target 2 (b), target 3 (c), target 4 (d), target 5 (e), and target 6 (f) are shown, respectively. For each target, DVHs from plans using tangential photon beam (PB), electron beam (EB), high‐dose‐rate (HDR) brachytherapy with surface applicator, intensity‐modulated radiation therapy (IMRT), and volumetric‐modulated arc therapy (VMAT) are shown. The doses delivered to the skin in high‐dose regions are higher than the others when using the PB technique.

**Figure 7:**
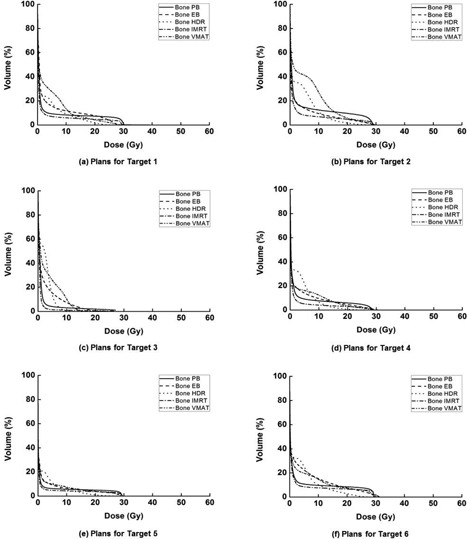
The dose‐volume histograms (DVHs) of the bone from the treatment plan for target 1 (a), target 2 (b), target 3 (c), target 4 (d), target 5 (e), and target 6 (f) are shown, respectively. For each target, DVHs from plans using tangential photon beam (PB), electron beam (EB), high‐dose‐rate (HDR) brachytherapy with surface applicator, intensity‐modulated radiation therapy (IMRT), and volumetric‐modulated arc therapy (VMAT) are shown. The doses delivered to the bone in high‐dose regions are higher than the others when using the PB technique. In the case of the HDR plans, the doses in the low‐dose region tend to be higher than the other techniques.

Dose‐volume histograms for each single target, foot OAR, skin, and bone are illustrated in Fig. 4, Fig. 5, Fig. 6, and Fig. 7, respectively. The high‐dose‐rate brachytherapy technique with surface applicator showed the worst target homogeneity. Consequently, the maximum doses to target were the highest in HDR compared to the other techniques. The technique using VMAT showed better target coverage and homogeneity than the other techniques. For PB plans, the doses to the foot OAR, skin, and bone in high‐dose regions were higher than the other techniques, while, in low‐dose regions, the doses of PB plans were lower than the other techniques. In the case of HDR plans, the doses to the foot OAR in high‐dose regions were lower than the other techniques, while doses to the foot OAR in low‐dose regions were higher than the other techniques.

Table 1 summarizes the averaged dosimetric results of the various techniques for single target. The value of HI of HDR was the highest with a value of 1.52. Plans using IMRT and VMAT showed the lowest HI with a value of 1.04. Conformity index was the highest in the IMRT plan with a value of 4.6, while the CI of the VMAT plan was closest to unity with a value of 0.8. In terms of target conformity and homogeneity, VMAT showed better results that the others.

**Table 1:** Dosimetric results of treatment plans for single target.

*Treatment Type*	*PB*	*EB*	*HDR (FF)*	*IMRT*	*VMAT*
MU or total dwell time (N/A or sec)	427±83	323±12	434±111	574±128	591±28
Target mean dose (Gy)	29.3±0.5	30.4±0.9	34.7±1.4	29.1±0.5	29.1±0.1
Target dose maximum (Gy)	30.3±1.0	32.1±1.2	56.2±3.5	30.1±0.6	30.3±0.2
Target dose minimum (Gy)	27.5±0.7	25.6±1.7	24.0±1.7	27.3±0.7	26.8±0.5
CI	1.97±0.98	2.39±0.51	1.60±0.48	4.60±4.58	0.80±0.11
HI	1.05±0.03	1.11±0.04	1.52±0.07	1.04±0.02	1.04±0.01
$V_{105\%}$ of target (%)	1.8±4.0	22.9±32.7	70.8±11.2	0.0±0.0	0.0±0.0
$V_{120\%}$ of target (%)	0.0±0.0	0.0±0.0	34.2±12.3	0.0±0.0	0.0±0.0
Mean dose of foot OAR (Gy)	2.5±0.7	2.7±1.0	3.6±0.7	1.7±0.5	2.9±0.7
$D_{50\%}$ of foot OAR (Gy)	0.3±0.1	0.3±0.2	1.9±0.5	0.2±0.1	0.3±0.2
$V_{20\text{Gy}}$ of foot OAR (%)	6.4±2.0	4.9±2.5	2.2±1.4	4.0±1.5	3.2±1.7
$V_{10\text{Gy}}$ of foot OAR (%)	7.9±2.5	9.6±4.1	7.6±3.2	5.1±1.8	10.6±2.7
Maximum dose to the skin (Gy)	30.3±1.1	26.8±2.7	35.7±4.5	31.1±1.5	28.8±0.4
Mean dose to the skin (Gy)	1.7±0.5	1.0±0.6	0.8±0.4	1.5±0.4	1.6±0.5
Maximum dose to the bone (Gy)	32.6±6.3	31.0±1.9	27.3±5.8	30.6±1.1	28.4±3.2
Mean dose to the bone (Gy)	2.5±0.8	2.9±0.7	2.0±1.9	1.7±0.6	3.5±1.2
Gradient measure (cm)	0.9±0.2	1.1±0.2	1.0±0.2	2.2±0.7	1.5±0.1
Integral dose (Gy‐cc)	4121±1121	4043±1601	5348±1202	3056±952	4694±1161

PB = photon beam; EB = electron beam; HDR = high‐dose‐rate brachytherapy; FF = Freiburg flap applicator; IMRT = intensity‐modulated radiation therapy; VMAT = volumetric‐modulated arc therapy; MU = monitoring unit; CI = conformity index; HI = homogeneity index; $V_{n\%}$ (n Gy) = volume irradiated at least n% (n Gy) of prescription dose; $D_{n\%}=\text{the minimum dose delivered to n}\%\;\text{volume of certain structure}$; OAR = organ at risk; foot OAR = foot subtracted by target.

The mean dose to the foot OAR in plans using PB, EB, HDR, IMRT, and VMAT were 2.5 Gy, 2.7 Gy, 3.6 Gy, 1.7 Gy and 2.9 Gy, respectively, with the IMRT plan demonstrating the lowest value. The value of $D_{50\%},V_{10\text{Gy}}$, and integral dose of the foot OAR was also lowest in the IMRT plan with a value of 0.2 Gy, 5.1%, and 3056 Gy‐cc, respectively. The mean dose to the skin and the maximum dose to the bone were lowest in the HDR plan. The maximum dose to the skin was lowest in the EB plan. The mean dose to the bone was lowest in the VMAT plan. In terms of normal tissue sparing, IMRT generally showed better results than the others.

### B. Dose‐volumetric results for multitarget

**Figure 8:**
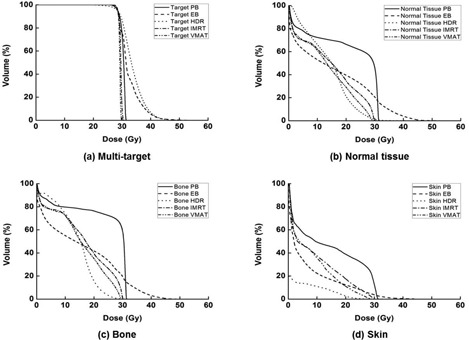
The dose‐volume histograms (DVHs) of multitarget (a), normal tissue (b), bone (c), and skin (d) from plans using 4‐box photon beam with multileaf collimator (PB), electron beam (EB), high‐dose‐rate (HDR) brachytherapy, intensity‐modulated radiation therapy (IMRT), and volumetric‐modulated arc therapy (VMAT) are shown. When using the EB technique, the maximum dose to normal tissue is higher than the other techniques. In the high‐dose region, the EB plan shows larger irradiated volume of normal tissue than others, while the HDR plan shows largest irradiated volume of normal tissue in the low‐dose region. When using the HDR technique, the doses to the skin in the intermediate and low‐dose regions were lowest.

**Figure 9:**
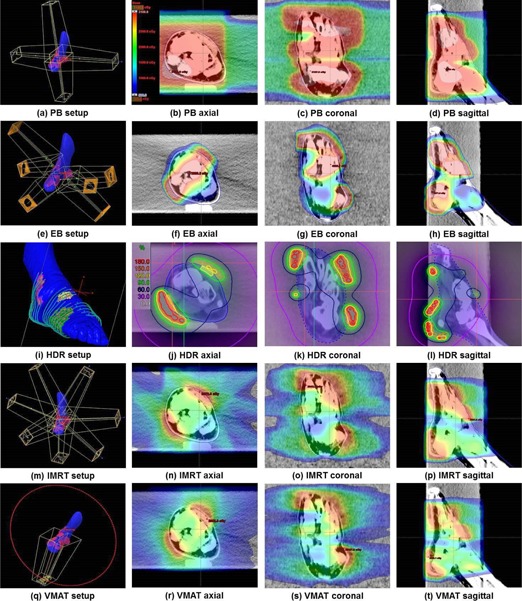
The beam setup and the dose distributions of treatment plans for multitarget using 4‐box photon beam with multileaf collimator (PB), electron beam (EB), high‐dose‐rate (HDR) brachytherapy, intensity‐modulated radiation therapy (IMRT), and volumetric‐modulated arc therapy (VMAT) are shown. The beam setup of PB (a), EB (e), HDR (i), IMRT (m), and VMAT (q) are shown. The axial dose distribution of PB (b), EB (f), HDR (j), IMRT (n), and VMAT (r) are shown. The sagittal dose distribution of PB (d), EB (h), HDR (l), IMRT (p), and VMAT (t), as well as the coronal dose distribution of PB (c), EB (g), HDR (k), IMRT (o), and VMAT (s), are also shown.

The DVHs of the multitarget and OARs are illustrated in Fig. 8. The dosimetric results from various treatment plans are summarized in Table 2. The beam setup and the dose distributions of each technique are shown in Fig. 9. Target dose maximum was highest in the HDR plan with a value of 56.4 Gy, while target dose minimum of the HDR plan was the lowest with a value of 19.2 Gy. Therefore, the HI of the HDR plan was the highest with a value of 1.43, which indicates the worst homogeneity in the target. The values of CI and HI for VMAT plan were closest to unity with values of 0.84 and 1.04, respectively. The values of $V_{105\%}$ of target in the PB, EB, HDR, IMRT, and VMAT plans were 0%, 50.1%, 70.2%, 14.2%, and 0%, respectively. In terms of the dose volumetric quality for the multitarget, the VMAT plan showed the better results than the other techniques. The plan with EB showed the worst target conformity, which means the high‐dose irradiation of normal tissue adjacent to the target was severe in the EB plan.

**Table 2:** Dosimetric results of treatment plans for multitarget.

*Treatment Type*	*PB*	*EB*	*HDR (FF)*	*IMRT*	*VMAT*
MU or total dwell time (N/A or sec)	342	1825	1772	1420	986
Target mean dose (Gy)	30.3	32.3	33.7	29.5	29.2
Target dose maximum (Gy)	31.5	47.9	56.4	30.8	30.5
Target dose minimum (Gy)	25.4	24.3	19.2	25.7	25.8
CI	3.99	5.08	1.38	1.95	0.84
HI	1.10	1.36	1.43	1.06	1.04
$V_{105\%}$ of target (%)	0.0	50.1	70.2	14.2	0.0
$V_{120\%}$ of target (%)	0.0	13.8	25.2	0.0	0.0
Mean dose of foot OAR (Gy)	21.3	14.6	14.2	14.3	13.0
$D_{50\%}$ of foot OAR (Gy)	29.6	10.5	14.9	15.0	13.2
$V_{20\text{Gy}}$ of foot OAR (%)	66.4	37.4	21.2	34.7	29.0
$V_{10\text{Gy}}$ of foot OAR (%)	72.4	50.8	65.7	62.5	61.0
Maximum dose to the skin (Gy)	31.3	52.8	36.4	30.2	29.6
Mean dose to the skin (Gy)	13.8	6.6	1.5	8.9	7.8
Maximum dose to the bone (Gy)	31.4	51.8	34.8	30.7	30.2
Mean dose to the bone (Gy)	17.9	16.4	15.2	14.0	13.1
Gradient measure (cm)	4.3	1.2	2.9	3.6	3.3
Integral dose (Gy‐cc)	30989	21571	21113	22224	20571

PB = photon beam; EB = electron beam; HDR = high‐dose‐rate brachytherapy; FF = Freiburg flap applicator; IMRT = intensity‐modulated radiation therapy; VMAT = volumetric‐modulated arc therapy; MU = monitoring unit; CI = conformity index; HI = homogeneity index; $V_{n\%}$ (n Gy) = volume irradiated at least n% (n Gy) of prescription dose; $D_{n\%}=\text{the minimum dose delivered to n}\%\;\text{volume of certain structure}$; OAR = organ at risk; foot OAR = foot subtracted by target.

The mean dose to the foot OAR of the VMAT plan was the lowest with values of 13.0 Gy. The values of $D_{50\%},\;V_{10\text{Gy}}$, and GM were lowest in the EB plan with values of 10.5 Gy, 50.8%, and 1.2 cm, respectively. The irradiation of normal tissue by relatively low dose was smaller in the EB plan compared to the others. The value of $V_{20\text{Gy}}$ was lowest in the HDR plan, with a value of 21.2%. The plan using the PB technique showed the highest mean dose, $D_{50\%}$, $V_{20\text{Gy}}$, $V_{10\text{Gy}}$, GM, and integral dose for the foot OAR with values of 21.3 Gy, 29.6 Gy, 66.4%, 72.4%, 4.3 cm, and 30989 Gy‐cc, respectively. The integral doses of the PB, EB, HDR, IMRT, and VMAT plans were 30989 Gy‐cc, 21571 Gy‐cc, 21113 Gy‐cc, 22224 Gy‐cc, and 20571 Gy‐cc, respectively, with the lowest value of the integral dose found in the VMAT plan. The maximum dose to the skin (29.6 Gy) and bone (30.2 Gy), as well as the mean dose to the bone (13.1 Gy), were also lowest in the VMAT plan. The mean dose to the skin was lowest in the HDR plan with a value of 1.5 Gy.

## IV. DISCUSSION

This study investigated the feasibility of adopting VMAT techniques to treat cutaneous KS developed on the skin of the foot at the planning level. The dosimetric results of treatment plans using conventional techniques to treat multifocal skin disease were compared with the results of the VMAT plan. Three‐dimensional conformal radiation therapy technique, electron beam technique, high‐dose‐rate brachytherapy with surface applicator, and IMRT were all compared with VMAT in terms of target conformity, target homogeneity, and the sparing of normal tissue, while maintaining the same target coverage.

For the single‐target plans, VMAT didn't always demonstrate superiority to the other techniques in terms of dose‐volumetric results. Thus the most suitable technique for single‐target plans was dependent on the target location and shape. However, the plan using the VMAT technique showed better quality for the multitarget in terms of target conformity, homogeneity, the mean dose to the normal tissue, the mean dose to the bone, and the maximum dose to the skin and bone than did the other techniques, while maintaining the same target coverage. Compared with multi‐isocentric summed plans using electron beams, VMAT was superior in terms of the sparing of resources such as treatment time and labor intensity, as well as plan quality for the target. In addition, the mean dose to the foot OAR and the integral dose were lower in the VMAT plan than in the EB plan. Single‐isocentric plans using PB technique were not dosimetrically advantageous compared with VMAT plans even though the consumption of resources for each during treatment was comparable. Treatment plans using the HDR brachytherapy technique with the FF applicator showed better values for $V_{20\text{Gy}}$ and GM than did VMAT plans. However, with the exception of $V_{20\text{Gy}}$ and GM, VMAT plans always demonstrated better values of dosimetric indicators than did the HDR plans. In the case of IMRT, VMAT was always superior to the IMRT even though the differences were not large. The cutaneous KS is a multifocal disease and, therefore, the dosimetric results from the plans for the multitarget better reflect reality. For the treatment of cutaneous KS developed on the skin of the foot, VMAT seems to be a feasible treatment modality.

A previous study has demonstrated the effectiveness of VMAT on cutaneous KS of the lower extremities, including feet with a bolus.^(15)^ The dosimetric results of the VMAT plans were compared with those of electron beam therapy as a conventional treatment modality. Similar to our results, it was shown that VMAT plans were superior to electron beam therapy in terms of both plan quality and consumption of resources. The difference between our study and previous studies was that the conventional technique comparison groups were extended to include the HDR with surface applicator and also the photon beam technique (3D CRT and IMRT).

To treat multifocal skin disease of the foot, the VMAT technique could be considered better than either the EB or HDR brachytherapy techniques from the practical point of view. The previous study reported that the delivery of VMAT was 2.5–3.4 times faster compared to EB technique.^(15)^ For HDR brachytherapy with the surface applicator, it has been shown that a bolus of at least 10 cm should be attached above the applicator to provide full‐scatter conditions when using the AAPM TG‐43 protocol without inhomogeneity correction.^(17)^ This could make the daily patient setup more difficult. Even though the VMAT needs pretreatment quality assurance, which is an additional consumption of resources compared to the other techniques such as PB, EB, and HDR, at least VMAT could reduce the resources needed during treatment.

A limitation of this study is that the investigation was performed only at plan level with virtual targets. Even though measurements could make this study more solid, we couldn't perform measurements and they will be performed in the future. However, various studies have already investigated the accuracy of dose calculation algorithms by comparison with measurements and through Monte Carlo simulations, including situations with inhomogeneity.^24^, ^25^, ^26^, ^27^, ^28^, ^29^, ^30^, ^31^, ^32^, ^33^, ^34^, ^35^, ^36^ Based on previous studies of commercial dose calculation algorithms, the dosimetric information from the TPS used in this study seems to be reliable. Even though this study doesn't include measurements, planning results should give sufficient information about the different characteristics of the different treatment modalities for skin cancer of the foot. The targets in this study were delineated virtually. Even though those were not real, since KS could be developed anywhere in the skin region, we thought the virtual targets could simulate the real situations properly. The other limitation is that this study was performed with only a single case. Therefore, the statistical significance was not calculated. Future work could involve collecting numerous patient cases of cutaneous KS of the foot, which will allow statistical analysis to be performed.

## V. CONCLUSIONS

The feasibility of using VMAT for the treatment of Kaposi's sarcoma developed on the skin of foot was demonstrated. Treatment plans using VMAT techniques were superior to plans using conventional technique such as PB, EB, HDR with surface applicator, and IMRT for multifocal targets in terms of both plan quality and treatment efficiency. It is feasible to use VMAT for the multifocal skin disease including cutaneous KS of the foot.
